# General Practitioners’ Awareness and Perception of Current Pneumococcal Vaccination for Adult Patients with Known Risk Factors in Switzerland: Evidence from a Survey

**DOI:** 10.3390/vaccines11061101

**Published:** 2023-06-15

**Authors:** Sandro Tiziano Stoffel, Matthias Schwenkglenks, Thomas Mutschler

**Affiliations:** 1 Research Department of Behavioural Science and Health, UCL, London WC1E 6BT, UK; 2 Institute of Pharmaceutical Medicine (ECPM), University of Basel, CH-4056 Basel, Switzerland; 3 Medical Affairs, MSD, CH-6005 Lucerne, Switzerland

**Keywords:** invasive pneumococcal disease, pneumococcal vaccine, survey, physician, general practitioner, knowledge, attitudes, vaccination

## Abstract

In Switzerland, the National Immunization Advisory Group (NITAG) has formulated recommendations for pneumococcal vaccination among adult risk patients. Little is known about general practitioners’ (GPs’) perception, knowledge, and implementation of these recommendations. Therefore, we investigated GPs’ awareness and drivers of and barriers to pneumococcal vaccination using a cross-sectional web-based survey of GPs. Of the 300 study participants, 81.3% were aware of the recommendations for vaccinating at-risk adult patients, but only 42.7% were aware of all risk groups. The recommendations were perceived by 79.7% as slightly to very complex. Most GPs (66.7%) had good arguments to convince patients to get vaccinated, but only 41.7% reported recognizing patients at risk for pneumococcal disease, and only 46.7% checked their patients’ vaccination status and proposed vaccination if needed. The main reasons for not vaccinating were patients’ refusal (80.1%), lack of reimbursement by the health insurance (34.5%), patients’ fear of side effects (25.1%), and lack of regulatory approval despite the NITAG recommendations (23.7%). Most (77.3%) agreed that the treating chronic disease specialist should recommend the vaccination and 94.7% believed that adult-risk patients would not be aware of their need for pneumococcal vaccinations. Optimal implementation of the recommendations will require addressing knowledge gaps and reported barriers.

## 1. Introduction

Pneumococcal infection is a disease caused by the Gram-positive bacterium *Streptococcus pneumoniae*, also known as pneumococcus, and can lead to serious invasive disease, as well as a milder but more common non-invasive disease [1,2]. Pneumococcal-related diseases, including pneumonia, bacteraemia, meningitis, and otitis media, are among the most frequent vaccine-preventable infectious diseases and are associated with considerable morbidity and mortality [2,3]. Infants and children aged <2 years, older adults, immunocompromised individuals, and people with chronic conditions are particularly susceptible to pneumococcal disease [1].

Pneumococcal conjugate vaccines (PCVs) are an effective way to prevent pneumococcal disease and thereby reduce the burden of disease and cost [4,5]. In Switzerland, PCV13 vaccination against pneumococcal disease is recommended by the NITAG (National Immunization Advisory Group), locally called EKIF (Eidgenössische Kommission für Impffragen), as a basic vaccination for children up to 5 years. Furthermore, EKIF recommends PCV13 for all individuals with known risk factors for invasive pneumococcal disease [6]. While in children aged 2 years the pneumococcal vaccination rates (three doses) are high and reached 84% in 2017–2019 [7], only a few studies have investigated the vaccination coverage rate of adult risk patients in Switzerland [8,9,10]. A recent study looking at PCV13 vaccination-based immunogenicity in inflammatory bowel disease (IBD) patients suggested that only 10 out of 538 (1.9%) of approached IBD patients had been vaccinated against pneumococcal disease [8]. Similarly, a study examining vaccination records found that only 2.7% of individuals reporting no health predisposition were vaccinated, while those with asthma or chronic pulmonary disease, or a compromised immune system, had higher coverages of 14.8% and 27.1%, respectively [9]. The highest vaccine coverage (32.5%) was observed among adult patients with axial spondyloarthritis (axSpA) treated with biological drugs [10].

These findings are similar to those reported by international studies, which also suggest low uptake [11,12,13,14,15,16].

In France, only 4–6% of adult risk patients with COPD, diabetes mellitus, or congestive heart disease were vaccinated against pneumococcal disease by their physicians [17,18]. In Germany, vaccination rates varied between 11.5% and31% for older adults (>60 years) [19]. A higher vaccination uptake of 49% was observed for adult patients with autoimmune disorders under biological therapy in the French overseas department Réunion [20]. The low uptake has been explained by the patients’ and physicians’ low awareness of the recommendations for pneumococcal vaccination [21,22,23]. These studies from Europe and the United States indicated that physicians lacked awareness of the recommendations or struggled with them [21,22,23]. As physicians’ recommendations are a major reason for getting vaccinated, it is important to understand their vaccination-related knowledge and perceived barriers [20,22,24].

In Switzerland, groups at higher risk for pneumococcal diseases are generally well-defined in the annual National Immunization Plan (NIP 2021) [6]. However, the definitions in the recommendations are very detailed so that even infectious disease specialists may struggle to be aware at which disease stage every given patient group is recommended to receive pneumococcal vaccination. For example, there is a recommendation for diabetes patients, but only when diabetes is associated with heart, lung, or kidney disease. The recommendation for COPD patients is limited to those with stage 3 or 4 according to the GOLD classification.

Currently, it is unclear how well these recommendations are known among GPs and how they are implemented. Additionally, vaccination coverage rates (VCRs) for pneumococcal vaccination of at-risk patients are not available, and hence, it is not known what proportion of people are vaccinated against pneumococcal disease. A further potential barrier to adult pneumococcal vaccination is the unique Swiss situation with NITAG recommending PCV13 for at-risk adults despite the lack of approval from the regulatory authority Swissmedic and reimbursement from the statutory health insurance [17]. This study aims to investigate the drivers of and barriers to pneumococcal vaccination in Swiss GPs.

## 2. Materials and Methods

### 2.1. Study Population and Design

A cross-sectional web-based survey with almost exclusively closed-type questions was developed and conducted on a randomized, layered sample of GPs in the German- and French-speaking parts of Switzerland. Data collection was conducted over 12 weeks in autumn 2021 (September to November). A total of 300 study participants were recruited by a survey vendor specializing in Swiss healthcare professional recruitment for surveys (QualiPro) from their existing database. Due to the limited physicians in the Italian-speaking part of Switzerland (Ticino), GPs were recruited according to their representation in the German- and French-speaking parts of Switzerland (231 German-spoken and 69 French-spoken physicians).

To qualify for the study, GPs had to have been practising in an office-based or ambulatory setting within Switzerland for 2–35 years and consent to participate in the study after being presented with its topic and the safeguards to ensure the anonymity of participants.

All survey questions are presented in the Supplementary Materials. The study was preregistered on Open Science Framework (OSF) before the start of the data collection.

### 2.2. Measures

#### 2.2.1. Self-Reported Awareness of the Recommendations for Vaccinating Adult Patients at Known Risks

Consenting GPs were provided with information about the current NITAG recommendation and asked whether they recognised the NITAG recommendation on vaccinating adults (“Before today, were you aware of the recommendation for vaccinating adults with known risk factors for invasive pneumococcal disease?”) and whether they recalled all risk groups for which the PCV13 vaccination is recommended (“Were you aware of all the risk patients, for which the pneumococcal vaccine is recommended?”) [25]. Both questions featured dichotomous (Yes/No) response options. Participants who stated that they did not know all risk groups were asked to indicate the included chronic diseases, immune disorders, neoplasia, and transplantations they had not been aware of.

#### 2.2.2. Perception of the Recommendations and Pneumococcal Vaccination

GPs’ perceptions of the recommendations were assessed through two questions on the perceived complexity of the recommendations, using a fully labelled 4-point Likert scale and their agreement with them (Yes/No). Beliefs about the pneumococcal vaccination were measured through six statements featuring fully labelled 7-point Likert scales (1 = strongly disagree, 7 = strongly agree): “Adult risk group patients are aware that they need a pneumococcal vaccination”, “The treating specialist for the risk factors/chronic conditions should recommend the GP to vaccinate the risk patients”, “I recognize patients at risk for pneumococcal disease”, “I check the vaccination status of risk patients and propose a pneumococcal vaccination, if needed”, “I have good arguments to convince adult risk patients of the importance of the pneumococcal vaccination”, and “Pneumococcal vaccination for adult risk groups is not important, since it is not covered by the basic health insurance”. The general perception of the pneumococcal vaccination was assessed through the questions: “How important do you think it is for adults with known risk factors for invasive pneumococcal disease to get COVID-19/pneumococcal/herpes zoster/influenza vaccination?”. All four questions used the same fully labelled five-point Likert scale (1 = not important, 5 = very important) and were presented in random order and were adapted from Klett-Tammen and colleagues [23].

#### 2.2.3. Perception of Pneumococcal Disease

General beliefs and perceptions about pneumococcal infections were measured by eight statements adapted from a recent study on varicella vaccination by Lienert and colleagues [26]: “The pneumococcal disease has a mild disease course for adults with known risk factors”, “The risk of pneumococcal disease is low even for adults with risk factors”, “Pneumococcal vaccination should mainly target children and not adults because of herd immunity”, “Pneumococcal disease can cause considerable morbidity and mortality”, “I am worried about the potential side effects of the pneumococcal vaccine for adults with known risk factors”, “I think that the pneumococcal disease is serious enough for adults with known risk factors to justify vaccination”, “Pneumococcal vaccination of risk groups is even more important during the COVID-19 “pandemic”, and “Simplification of vaccination guideline for pneumococcal vaccination in adults would result in more risk patients being vaccinated.” Each statement was measured with a fully labelled 7-point Likert scale (1 = strongly disagree, 7 = strongly agree).

#### 2.2.4. Perceived Barriers to Pneumococcal Vaccination

Study participants were asked to indicate possible reasons for not advising pneumococcal vaccinations despite NITAG recommendations from a pre-specified list of 17 options presented in random order and adapted from Klett-Tammen and colleagues [23]. GPs were instructed to select all reasons they deemed relevant to them (see Supplementary Materials). One option stated the absence of specific reasons. Space was additionally provided for non-listed barriers to be added in a free-text format. These were subsequently coded and added to the overall list of barriers.

#### 2.2.5. GPs’ Demographic and Professional Characteristics

GPs were asked to provide their age category, gender, and years of experience as active GPs. Participants were also asked to provide details of the region in Switzerland and the language in which they practised mainly. Additionally, six questions from the 5C measure of psychological antecedents of vaccination were included to measure potential mediating effects [27]. The questions featured 7-point fully labelled Likert scales [0;6] and were combined into two constructs on confidence in vaccination and public authorities (confidence), and decision-making style and information-seeking behaviour (calculation) with scores between 0 and 18 each. A higher score indicates a higher level of trust and a more rational decision-making process regarding vaccinations.

#### 2.2.6. Previous Experience Vaccinating Adult Patients with Known Risks

Previous experience of vaccinating adult patients with known risk factors was assessed through four questions that asked GPs about the number of patients with known risk factors, whether they had personally vaccinated adult risk patients, and the percentage of their patients vaccinated against pneumococcal disease.

### 2.3. Sample Size and Statistical Analysis

Due to the exploratory nature of the study, no formal sample size was calculated beforehand. Descriptive statistics were calculated for questions on the perception of the recommendations, vaccination, infection, and barriers to vaccination. These results were reported using percentages. Binary and ordinal logistic regression analyses were used to assess multivariable associations between awareness of recommendations or risk groups, and GPs’ demographic and professional characteristics, general attitudes towards vaccination, previous experience with PCV13, and also the number of patients with known risk factors they had treated in the last year. The results of the regressions were reported using adjusted odds ratios (aORs) and 95% confidence intervals (CIs). Due to low frequencies in some answer categories, the 26 cantons were reclassified into seven major statistical regions of Switzerland: Lake Geneva region, Espace Mittelland, northwest Switzerland, Zurich region, eastern Switzerland, central Switzerland, and Ticino (details are shown in Table S1 in the Supplementary Materials) [28]. While the canton Ticino would be covered in the region Ticino, there were no observations given the focus on French-speaking and German-speaking Switzerland. Thus, this region was excluded from the analysis. All analyses were performed using Stata^®^ (version IC 16.0).

## 3. Results

### 3.1. Population Characteristics

Of 4607 invitees, 624 (13.5%) GPs responded to the e-mail invitation during the data collection period of 12 weeks. Of those, 387 (62.0%) started the survey, 38 did not complete it even after several reminder e-mails and phone calls, and 49 were excluded because they did not qualify. In total, 300 GPs completed the survey; this equates to a response rate of 6.5% (see Figure S1 in Supplementary Materials for a patient flowchart). Their main characteristics are described in Table 1.

Most participants were male (*n* = 179, 59.7%), practising in Zurich or Espace Mittelland (*n* = 123, 41%), had up to 15 years of practice experience (*n* = 147, 49%) and had between 21 and 50 patients with known risk factors (*n* = 105, 35.0%). Attitudes towards vaccination were generally positive with median confidence and calculation scores of 15 out of 18 each (see Figure S2 in Supplementary Materials for the distribution of the scores).

### 3.2. Awareness of the Recommendations for Vaccinating Adult Patients at Known Risks

Of the 300 study participants, 81.3% (*n* = 244) were aware of the recommendations for vaccinating adult patients at known risks, but only 42.7 % (*n* = 128) were aware of all risk groups. Table 2 outlines regression-adjusted results, which showed that GPs were less likely to be aware of the recommendations if they practised in eastern Switzerland and Espace Mittelland instead of the Lake Geneva region (88.2% vs. 70.0%; aOR 0.12, 95% CI 0.02–0.76, *p* = 0.025). Additionally, GPs with fewer years of work experience were more likely to recall the vaccination recommendations. Results were different for knowing all the risk groups. GPs were significantly more likely to know all risk groups if they had more than 100 patients with known risk factors (aOR 3.14, 95% CI 1.39–7.11, *p* = 0.006) and were more engaged in extensive information searching (calculating) and rational in deciding about vaccinations (aOR 1.15, 95% CI 1.03–1.28, *p* = 0.013).

**Figure 1:**
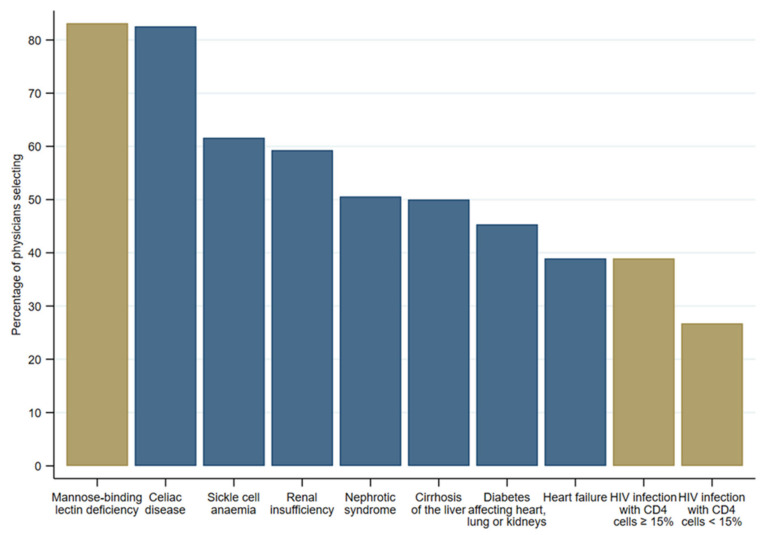
Top 10 most frequently mentioned risk groups physicians were not aware of (*n* = 172).

The five least known risk groups were patients with Mannose-binding lectin deficiency (*n* = 143, 83.1%), celiac disease (*n* = 142, 82.6%), sickle cell anemia (*n* = 106, 61.6%), renal insufficiency (*n* = 102, 59.3%), and nephrotic syndrome (*n* = 87, 50.6%). Figure 1 shows that seven out of the ten least known risk groups belonged to the group of chronic diseases (in blue) and three to immune disorders (in brown).

See Figure S3 in the Supplementary Materials for the frequencies of all risk groups.

### 3.3. Agreement and Perception of the Current Vaccination Recommendation

Almost all GPs agreed with the recommendations (97.7%; *n* = 293) and also stated to be following them (98.3%; *n* = 295). Reasons for not agreeing with the recommendations were that the risk groups were perceived as too broad and that the statutory health insurance would not cover the vaccination. 

The NITAG recommendations were perceived by 20.3% (*n* = 61) as not complex at all, by 45.3% (*n* = 136) as slightly complex, by 28.0% (*n* = 84) as moderately complex, and by 6.3% (*n* = 19) as very complex. An ordered logistic regression did not reveal any statistically significant associations with GPs’ characteristics (see Table S2 in Supplementary Materials).

### 3.4. Perception of Pneumococcal Vaccination

As shown in Table 3, 41.7% of GPs reported that they would recognize patients at risk for pneumococcal disease (strongly agree or moderately agree), whereas 77.3% strongly or moderately agreed that the treating chronic disease specialist should recommend the GP to vaccinate their risk patient. A total of 66.7% of GPs strongly or moderately agreed that they have good arguments to convince patients to get vaccinated: 46.7% strongly or moderately agreed that they check their patients’ vaccination status and propose vaccination if needed. Only 5.3% of GPs strongly or moderately agreed that adult patients with known risk factors would be aware of their need for pneumococcal vaccination, and even fewer (3.7%) agreed strongly or moderately that the vaccination would be not important because it is not covered by the basic health insurance (see Figure S4 in Supplementary Materials for the full distribution of responses).

### 3.5. Attitudes towards Pneumococcal Disease and Vaccination

Table 4 shows that the majority of the participating GPs strongly or moderately agreed that pneumococcal vaccination is even more important during the COVID-19 pandemic (68.3%) and that pneumococcal disease is serious enough to warrant vaccination (85.3%). A majority also believed that more patients would get vaccinated if the recommendations were simpler (59.7%) and that pneumococcal disease is a serious disease with considerable morbidity and mortality (72.7%). A small minority agreed with the statements that the disease has a mild course for adults with known risk factors (4.3%), that their risk of the disease is low (2.3%), that they are worried about the side effects of the vaccine (2.3%), and that only children should be vaccinated (2.3%, see Figure S5 in the Supplementary Materials for the full distribution of responses).

The ordered logistic regressions in Table 5 show that being calculating and engaging in extensive information searching was positively associated with believing that the treating specialist should recommend vaccination (aOR 1.12, 95% CI 1.03–1.23, *p* = 0.010), with the belief of having good arguments to convince patients of the importance of the vaccination (aOR 1.17, 95% CI 1.07–1.29, *p* = 0.001), checking vaccination status (aOR 1.18, 95% CI 1.08–1.29, *p* < 0.001), and recognizing patients at risk (aOR 1.23, 95% CI 1.14–1.35, *p* < 0.001).

**Figure 2:**
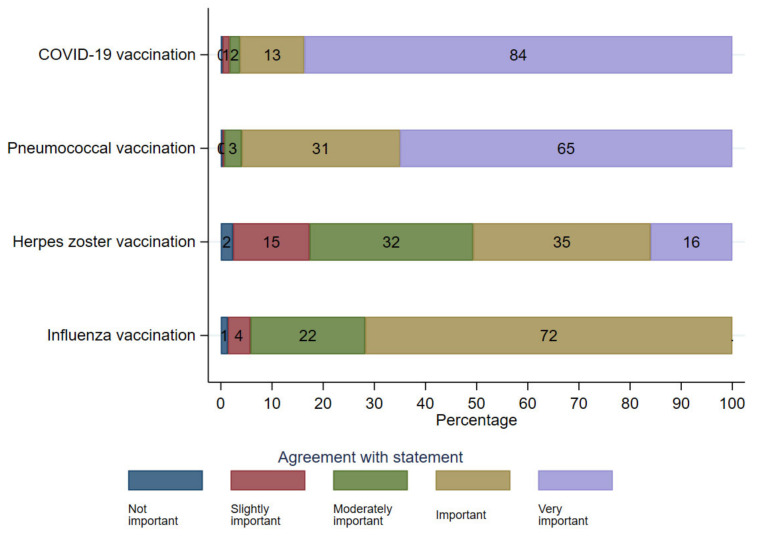
Perceived importance of different vaccinations for adults with known risk factors (*n* = 300).

Similarly, confidence in vaccination was positively associated with the belief that the treating specialist should recommend vaccination (aOR 1.15, 95% CI 1.06–1.24, *p* = 0.001) and the notion to have good arguments for vaccinating patients (aOR 1.15, 95% CI 1.06–1.25, *p* = 0.001). Having a large number of patients with known risk factors (more than 100) was positively associated with checking vaccination status (aOR 2.18, 95% CI 1.09–4.35, *p* = 0.027) and having 51–100 or more than 100 risk patients was positively associated with recognizing patients at risk (aOR 2.02, 95% CI 1.09–3.75, *p* = 0.026 and aOR 2.40, 95% CI 1.18–4.89, *p* = 0.016). The regressions for the attitudes towards pneumococcal infection are presented in Table S3 in Supplementary Materials. There were only a few statistically significant associations between the regions in which GPs practised and their perception of the course and risk of the disease. In comparison with other vaccinations, such as for COVID-19, herpes zoster, and influenza, GPs perceived the pneumococcal vaccination as the second most important. While 84% (*n* = 252) of participants stated that the COVID-19 vaccination is very important, 65% (*n* = 195) perceived the pneumococcal vaccination as very important (see Figure 2). 

### 3.6. Current Pneumococcal Vaccination Practice

**Figure 3:**
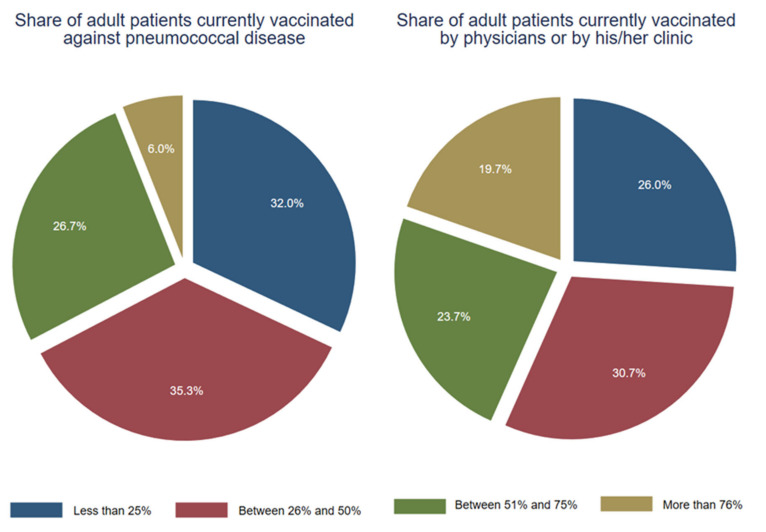
Vaccination of adult patients with known risk factors (*n* = 300).

Only a third (*n* = 98; 32.7%) of the GPs stated that more than half of their patients with known risk factors were currently vaccinated. Another third of GPs (*n* = 106, 35.3%) thought that around 26–50% of their patients were vaccinated. When asked who vaccinated these risk patients, 43.3% (*n* = 130) GPs responded that more than half of them were vaccinated by themselves or in their office (see Figure 3). 

The ordered logistic regression analysis shown in Table 6 indicates that GPs with longer practice experience (21–25 years, aOR 0.41 95% CI 0.17–0.99, *p* = 0.050) or who were practising in Espace Mittelland or eastern Switzerland (aOR 0.27 95% CI 0.09–0.80, *p* = 0.018 and aOR 0.13, 95% CI 0.04–0.51, *p* = 0.003) had lower proportions of patients vaccinated. GPs practising in northwest Switzerland or eastern Switzerland (aOR 0.19 95% CI 0.05–0.71, *p* = 0.013 and aOR 0.18, 95% CI 0.05–0.68, *p* = 0.011) had a lower proportion of their patients being vaccinated by themselves or their clinic than those practising in the Lake Geneva region.

Nevertheless, physicians who practise mainly in the French language had lower proportions of patients vaccinated by themselves or their practice than GPs who practised mainly in German (aOR 0.14 95% CI 0.05–0.42, *p* = 0.001). Finally, a higher number of patients with known risk factors (between 51 and 100) was positively associated with vaccination by the GP (aOR 2.05 95% CI 1.12–3.77, *p* = 0.021).

### 3.7. Reasons for Not Vaccinating

**Figure 4:**
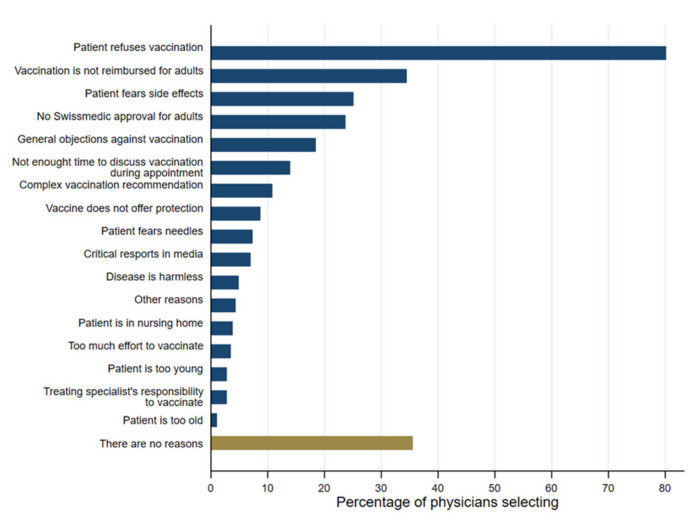
Barriers to vaccinating adult risk patients against pneumococcal disease (*n* = 300).

When asked for reasons for not vaccinating adults with known risk factors for invasive pneumococcal diseases in their clinic, most physicians (*n* = 230, 80.1%) stated that they would not vaccinate if their patients refused the vaccine. Other frequently mentioned reasons were that the vaccination is not reimbursed by the statutory health insurance (*n* = 99, 34.5%), that patients fear side effects (*n* = 72, 25.1%), and that the vaccination is not approved by Swissmedic for the studied patient group, which contrasts with the NITAG recommendation (*n* = 68, 23.7%). Figure 4 shows all the reasons for not vaccinating. Interestingly, more than a third of the GPs (*n* = 102, 35.5%) stated that there are no reasons for not vaccinating. 

## 4. Discussion

### 4.1. Summary

This study provides insights into Swiss GPs’ awareness of the current recommendations for vaccinating adult risk groups against pneumococcal infection. Importantly, most GPs recognize pneumococcal infection as serious and recommend their at-risk adults to get vaccinated. However, they struggle to identify certain chronic diseases as elevated risk factors for pneumococcal disease. Thus, increasing GP awareness could improve vaccination coverage and protect at-risk adults. Furthermore, GPs find recommendations complex and suggest simplification to increase vaccination rates. From the GPs’ perspective, vaccine safety (i.e., side effects) was not a perceived barrier. However, general refusal and fear of side effects of vaccination were the main reason on the patient’s side to refuse pneumococcal vaccination despite being at risk.

While GPs feel confident in convincing patients, they believe that their patients are unaware of their risks. Public campaigns could therefore raise awareness [22]. GPs see a lot of these risk patients in their offices and could be the main ones responsible for pneumococcal vaccination of adult-risk patients. However, they expect specialists to initiate recommendations. Thus, better awareness among specialists could boost vaccination rates, but achieving systematic recommendations by the specialists addressed to GPs is challenging.

### 4.2. Comparison with Existing Literature

The results of our study compare closely with previous GP studies. For example, the high perceived severity of pneumococcal infections and the belief that risk patients should be vaccinated have been observed in quantitative studies from Europe, Hong Kong, Lebanon, and the USA [21,22,24,29]. According to a Swiss qualitative study from 2012, GPs perceived the pneumococcal disease as potentially severe, but its vaccination as the least important vaccination in their daily practice, especially compared to influenza vaccination [30]. In our study, however, GPs perceived the vaccine as more important than the herpes zoster and the influenza vaccination.

High awareness of pneumococcal disease has been observed in previous studies from Europe and USA [21,22,31]. The European study featured a physician sample from 13 western European countries, including 100 Swiss GPs and 60 Swiss specialists, and investigated the awareness of pneumococcal infections [22]. There, GPs and specialists perceived pneumococcal infections to be less common compared to diseases like gastroenteritis and influenza, with GPs generally perceiving them as less frequent than specialists. Similarly, the Swiss study suggests that the GPs rarely ever experienced cases of severe pneumococcal disease in their daily work [30]. Note that these studies focused on disease awareness and vaccination decision, while our study focused on attitudes about the disease, vaccination, and recommendations. 

The only other study which investigated the perception of vaccination recommendations was conducted in the USA and found, similarly to our study, that the majority of GPs were aware of recommendations but believed that they should be simplified [21]. Note that in their study, knowledge was measured objectively through a series of case-based questions, while our study relies on self-reported knowledge. A systematic review has found that physicians do not appear to accurately self-assess their competencies [32]. The self-reported assessment of knowledge of the pneumococcal vaccination recommendation could have been subject to social desirability or superiority biases, which can affect the accuracy and reliability of the reported answers [33]. While the relatively low vaccination rate observed in this study is similar to those observed in other European studies using objective data on vaccination uptake from health registries [17,19,20], our study estimated the vaccination practice through questions about experience vaccinating their own patients.

Furthermore, our finding that GPs did not perceive the vaccine side effects, but the patients’ low awareness of the risk for pneumococcal disease as a vaccination barrier, has also been found in a large European cross-country survey [22]. Finally, the finding that GPs expected the treating specialists to recommend vaccination is consistent with a German study analysing advice-giving by GPs and physician assistants [23].

### 4.3. Strengths and Limitations

This was the first study that aimed to assess Swiss GPs’ knowledge of the NITAG recommendations for pneumococcal vaccination of adult risk groups and to measure attitudes towards pneumococcal infection and vaccination. The results may assist the Swiss NITAG in its evaluations of the formulation of the recommendations. Furthermore, the investigation of barriers to vaccination suggests that the lack of reimbursement by statutory health insurance and the lack of marketing approval by the regulatory authority are two major reasons for the low vaccination uptake. Similarly, the low awareness of the need for pneumococcal vaccination of risk groups could help health authorities to come up with interventions to increase vaccination rates. 

Our study has several limitations. Firstly, while our study featured a relatively large sample of GPs practising in Switzerland, the study sample was not fully representative of the Swiss population of GPs. Due to difficulties with recruitment, the French-speaking part of Switzerland was underrepresented. Furthermore, GPs from the Italian-speaking part of Switzerland, who make up 4.4% of all Swiss physicians, were not considered [26]. In line with other survey-based studies, our study may suffer from selection bias as GPs self-selected to participate in the survey [21,22,23,24,29]. Especially, given the relatively high scores for vaccination attitudes and the low response rate to this survey, we may have over-recruited GPs with more favourable views towards vaccination and higher awareness than Swiss GPs on average. Secondly, as in other survey-based studies, the outcomes of the study were based on GPs’ perceptions and feedback (i.e., stated preferences), which may have negatively affected the precision and validity of the results (i.e., recall and social desirability biases may have occurred). A further limitation of the study is that it relied on GPs’ self-assessment of their knowledge about the recommendation [32]. They may have responded inaccurately to be viewed in a positive light by the researcher [33]. However, we expect this risk of social desirability bias was at least partially mitigated by the anonymity of the survey. While the use of patient records would be better suited to study vaccination rates [20], it was not the goal of this study to assess vaccination rates. Thirdly, as the survey was cross-sectional, we do not know to what extent GPs’ knowledge of the recommendations and attitudes towards the vaccine developed over time. Future studies using longitudinal data could disentangle the nature of the relationship between awareness and actual vaccination administration in practice. Fourthly, our study was conducted during the COVID-19 pandemic in 2021. This may have impacted the response rate to the questionnaire and general attitudes towards vaccination, limiting the generalisability of the results [34]. Fifthly, while the analysis showed that patients’ refusal was the most frequently mentioned reason for not administering the vaccine, we did not assess different reasons for refusal. Future studies could investigate whether GPs’ decision to not vaccinate depends on the reason for the patient’s refusal. Finally, as our study was focused on the understanding and perception of the vaccination recommendations, we only focused on GPs’ perception of simplifying the recommendation as a potential intervention to increase vaccination uptake. Future research could test the acceptability of alternative interventions to increase uptake, such as strong recommendations from healthcare providers, reminder and recall efforts, and physician education [31,35,36]

## 5. Conclusions

In summary, the survey findings reported here indicate that GPs in our survey sample had a high awareness of the severity of pneumococcal disease in adult risk groups. They were aware of the NITAG recommendations for adults with elevated risk for pneumococcal disease and seemed convinced of the usefulness of pneumococcal vaccination. However, GPs had difficulties recognizing some risk groups in their daily practice and did not systematically vaccinate all risk patients. GPs expected that the specialists who treat conditions implying high risk should recommend the pneumococcal vaccination to the patient or the GP. Another perceived barrier to pneumococcal vaccination was the lack of regulatory approval and reimbursement of pneumococcal vaccination in at-risk adults. Based on these findings, optimal implementation of the recommendations will require addressing the knowledge gaps and reported barriers.

## Figures and Tables

**Table 1:** Background characteristics of participating physicians (*n* = 300).

Characteristics		N	(%)
**Gender**			
	Male	179	(59.7)
	Female	121	(40.3)
**Age**			
	40 years or younger	40	(13.3)
	41–45 years old	44	(14.6)
	46–50 years old	53	(17.7)
	51–55 years old	62	(20.7)
	56–60 years old	57	(19.0)
	60+ years old	44	(14.7)
**Practice experience**			
	2–10 years	85	(28.3)
	11–15 years	62	(20.7)
	16–20 years	45	(15.0)
	21–25 years	60	(20.0)
	26+ years	48	(16.0)
**Number of patients with known risk factors**			
	0–20	76	(25.3)
	21–50	105	(35.0)
	51–100	73	(24.3)
	More than 100	46	(15.3)
**Major region practising in**			
	Lake Geneva region	56	(18.7)
	Espace Mittelland	57	(19.0)
	Northwest Switzerland	39	(13.0)
	Zurich	66	(22.0)
	Eastern Switzerland	40	(13.3)
	Central Switzerland	42	(14.0)
**Main practising language**			
	German	231	(77.0)
	French	69	(23.0)
**General attitudes towards vaccination**			
	Confidence in vaccination [0;18] Median and SD	15	(2.90)
	Calculation [0;18] Median and SD	15	(2.69)

**Table 2:** Logistic regression on awareness of recommendation and adult risk patients (*n* = 300).

	Awareness of Recommendation [0;1]			Awareness of All Patient Groups at Risk [0;1]		
**Variable**	(%)	aOR	95% CI	(%)	aOR	95% CI
Overall	(81.3)			(42.7)		
**Gender**						
Male	(76.5)	Ref.		(44.1)	Ref.	
Female	(88.4)	1.792	0.858–3.743	(40.5)	0.748	0.437–1.281
**Age**						
40 or younger	(92.5)	Ref.		(37.5)	Ref.	
41–45	(93.2)	1.108	0.188–6.531	(36.4)	0.769	0.294–2.006
46–50	(81.8)	0.576	0.119–2.778	(34.0)	0.554	0.205–1.501
51-55	(75.8)	0.358	0.069–1.864	(43.6)	0.968	0.326–2.874
56–60	(68.4)	0.283	0.050–1.615	(40.4)	0.577	0.173–1.925
61 or older	(84.1)	0.781	0.109–5.583	(65.9)	1.424	0.377–5.384
**Practice experience**						
2–10 years	(92.9)	Ref.		(31.8)	Ref.	
11–15 years	(77.4)	0.246	0.072–0.846 *	(43.6)	1.711	0.763–3.836
16–20 years	(77.8)	0.474	0.114–1.964	(40.0)	1.442	0.530–3.921
21–25 years	(73.3)	0.341	0.081–1.436	(41.7)	1.512	0.542–4.219
26+ years	(79.2)	0.383	0.073–2.024	(64.6)	2.922	0.871–9.803
**Number of patients with known risk factors**						
0–20	(73.7)	Ref.		(36.9)	Ref.	
21–50	(82.9)	1.756	0.788–3.917	(36.2)	1.028	0.531–1.989
51–100	(89.0)	3.037	1.126–8.190 *	(48.0)	1.913	0.940–3.890
More than 100	(78.3)	1.154	0.438–3.043	(58.7)	3.142	1.390–7.105 **
**Major region practising in**						
Lake Geneva region	(83.9)	Ref.		(48.2)	Ref.	
Espace Mittelland	(80.7)	0.433	0.098–1.922	(43.9)	0.829	0.240–2.866
North-West Switzerland	(79.5)	0.189	0.027–1.330	(35.9)	0.555	0.118–2.616
Zurich	(84.9)	0.333	0.052–2.139	(43.9)	0.746	0.173–3.218
Eastern Switzerland	(70.0)	0.116	0.018–0.761 *	(45.0)	1.110	0.238–5.165
Central Switzerland	(85.7)	0.280	0.040–1.942	(35.7)	0.536	0.117–2.449
**Main practising language**						
German	(81.8)	Ref.		(41.6)	Ref.	
French	(79.7)	0.308	0.061–1.563	(46.4)	0.852	0.233–3.123
**Vaccination attitudes**						
Confidence		0.915	0.806–1.038		0.941	0.859–1.029
Calculation		1.109	0.977–1.257		1.147	1.030–1.277 *
N		300			300	

* *p* < 0.05; ** *p* < 0.01; aOR: adjusted odds ratio; CI: confidence interval.

**Table 3:** Perception and attitudes about pneumococcal vaccination (*n* = 300).

Questionnaire Item	Strongly or Moderately Disagree		Strongly or Moderately Agree	
	*n*	(%)	*n*	(%)
The treating specialist for the risk factors/chronic conditions should recommend the GP to vaccinate the risk patients.	4	(1.3)	232	(77.3)
I have good arguments to convince adult risk patients of the importance of the pneumococcal vaccination.	5	(1.7)	200	(66.7)
I check the vaccination status of risk patients and propose a pneumococcal vaccination if needed.	8	(2.7)	140	(46.7)
I recognize patients at risk for pneumococcal disease.	10	(3.3)	125	(41.7)
Adult risk group patients are aware that they need a pneumococcal vaccination.	177	(59.0)	16	(5.3)
Pneumococcal vaccination for adult risk groups is not important, since it is not covered by basic health insurance.	260	(86.7)	11	(3.7)

**Table 4:** Knowledge and attitudes towards pneumococcal disease in adult risk patients (*n* = 300).

Questionnaire Item	Strongly or Moderately Disagree		Strongly or Moderately Agree	
	*n*	(%)	*n*	(%)
I think that the pneumococcal disease is serious enough for adults with known risk factors to justify vaccination.	7	(2.3)	256	(85.3)
Pneumococcal disease can cause considerable morbidity and mortality.	18	(6.0)	218	(72.7)
Pneumococcal vaccination of risk groups is even more important during the COVID-19 pandemic.	11	(3.7)	205	(68.3)
Simplification of vaccination guidelines for pneumococcal vaccination in adults would result in more risk patients being vaccinated.	10	(3.3)	179	(59.7)
Pneumococcal disease has a mild disease course for adults with known risk factors.	237	(79.0)	13	(4.3)
The risk of pneumococcal disease is low even for adults with risk factors.	197	(65.7)	7	(2.3)
I am worried about the potential side effects of the pneumococcal vaccine for adults with known risk factors	215	(71.7)	7	(2.3)
Pneumococcal vaccination should mainly target children and not adults because of herd immunity.	219	(73.0)	7	(2.3)

**Table 5:** Ordinal logistic regressions on attitudes towards pneumococcal vaccination for adult patients with known risk.

	Treating Specialist Should Recommend the GP to Vaccinate the Risk Patients [1;7]		I Have Good Arguments to Convince Patients of the Importance of the Pneumococcal Vaccination [1;7]		I Check the Vaccination Status and Propose a Pneumococcal Vaccination if Needed [1;7]		I Recognize Patients at Risk for Pneumococcal Disease [1;7]		Patients Are Aware that They Need a Pneumococcal Vaccination [1;7]		Vaccination Is Not Important, as It Is Not Covered by Basic Health Insurance [1;7]	
**Variable**	aOR	95% CI	aOR	95% CI	aOR	95% CI	aOR	95% CI	aOR	95% CI	aOR	95% CI
**Gender**												
Male	Ref.		Ref.		Ref.		Ref.		Ref.		Ref.	
Female	0.708	0.442–1.137	0.803	0.501–1.287	0.992	0.632–1.558	0.864	0.541–1.380	0.844	0.535–1.331	0.732	0.433–1.240
**Age**												
40 or younger	Ref.		Ref.		Ref.		Ref.		Ref.		Ref.	
41–45	0.818	0.350–1.911	0.909	0.394–2.099	1.408	0.628–3.157	1.065	0.476–2.383	0.921	0.409–2.078	0.964	0.388–2.396
46-50	1.214	0.507–2.905	0.730	0.313–1.702	0.771	0.337–1.762	0.694	0.303–1.588	1.534	0.655–3.591	0.673	0.258–1.759
51–55	1.067	0.413–2.755	0.331	0.127–0.863 *	0.957	0.380–2.407	0.728	0.280–1.893	1.581	0.628–3.984	0.512	0.175–1.494
56–60	0.760	0.270–2.143	0.771	0.269–2.211	1.774	0.650–4.841	1.911	0.664–5.501	1.194	0.429–3.324	0.450	0.138–1.471
61 or older	0.694	0.210–2.294	0.872	0.269–2.832	2.960	0.971–9.019	3.806	1.171–12.370 *	1.856	0.575–5.991	0.346	0.086–1.386
**Practice experience**												
2–10 years	Ref.		Ref.		Ref.		Ref.		Ref.		Ref.	
11–15 years	0.974	0.481–1.972	0.759	0.384–1.499	1.371	0.700–2.682	0.853	0.430–1.692	1.237	0.634–2.413	1.153	0.531–2.501
16–20 years	0.552	0.235–1.297	1.832	0.767–4.377	0.820	0.352–1.912	0.796	0.331–1.912	0.646	0.282–1.479	1.727	0.653–4.562
21–25 years	0.752	0.318–1.779	1.562	0.653–3.736	0.637	0.274–1.479	0.514	0.212–1.245	1.076	0.468–2.473	1.762	0.654–4.747
26+ years	1.003	0.355–2.837	0.811	0.284–2.320	0.507	0.190–1.351	0.245	0.084–0.715 *	1.039	0.368–2.932	2.167	0.620–7.577
**Number of patients with known risk factors**												
0–20	Ref.		Ref.		Ref.		Ref.		Ref.		Ref.	
21–50	1.056	0.601–1.856	1.374	0.784–2.408	0.852	0.499–1.455	1.059	0.613–1.828	0.781	0.455–1.340	1.362	0.716–2.592
51–100	0.988	0.530–1.839	1.441	0.779–2.667	1.121	0.614–2.046	2.020	1.089–3.745 *	1.285	0.710–2.326	1.450	0.730–2.881
More than 100	1.020	0.495–2.102	1.925	0.959–3.864	2.180	1.093–4.348 *	2.400	1.178–4.888 *	0.975	0.492–1.934	1.026	0.445–2.364
**Major region practising in**												
Lake Geneva region	Ref.		Ref.		Ref.		Ref.		Ref.		Ref.	
Espace Mittelland	0.550	0.187–1.613	0.674	0.232–1.962	0.949	0.321–2.804	0.816	0.269–2.478	0.235	0.078–0.703 **	2.278	0.652–7.954
North–West Switzerland	0.427	0.109–1.670	0.423	0.111–1.617	0.517	0.135–1.989	0.377	0.097–1.474	0.315	0.083–1.198	1.037	0.224–4.800
Zurich	0.697	0.194–2.506	0.404	0.113–1.448	0.557	0.156–1.992	0.502	0.137–1.839	0.346	0.098–1.226	1.099	0.258–4.683
Eastern Switzerland	0.494	0.130–1.876	0.409	0.108–1.555	0.464	0.122–1.765	0.471	0.122–1.814	0.159	0.042–0.607 **	1.755	0.391–7.881
Central Switzerland	0.700	0.187–2.615	0.340	0.091–1.267	0.705	0.190–2.613	0.717	0.189–2.726	0.342	0.092–1.270	1.517	0.343–6.706
**Practising language**												
German	Ref.		Ref.		Ref.		Ref.		Ref.		Ref.	
French	1.020	0.332–3.131	0.446	0.144–1.374	0.763	0.247–2.357	1.043	0.329–3.302	0.353	0.111–1.128	0.639	0.186–2.204
**Vaccination attitudes**												
Confidence	1.149	1.062–1.242 **	1.151	1.064–1.246 **	1.009	0.934–1.091	0.988	0.914–1.068	0.967	0.897–1.042	0.919	0.842–1.002
Calculation	1.124	1.028–1.228 *	1.173	1.070–1.285 **	1.178	1.079–1.287 **	1.240	1.137–1.352 **	0.985	0.907–1.070	0.852	0.774–0.939 **
N	300		300		300		300		300		300	

* *p* < 0.05; ** *p* < 0.01; aOR: adjusted odds ratio; CI: confidence interval.

**Table 6:** Ordinal logistic regressions on vaccinating adult patients with known risk.

	Proportion of Patients Being Vaccinated [1;4]		Proportion of Those Patients Vaccinated by Physician or Clinic [1;4]	
**Variable**	aOR	95% CI	aOR	95% CI
**Gender**				
Male	Ref.		Ref.	
Female	0.912	0.576–1.444	0.996	0.630–1.574
**Age**				
40 or younger	Ref.		Ref.	
41–45	0.747	0.332–1.678	0.604	0.268–1.362
46–50	0.825	0.360–1.889	0.960	0.412–2.238
51–55	0.718	0.277–1.860	0.737	0.284–1.910
56–60	1.291	0.446–3.736	1.680	0.565–4.999
61 or older	1.928	0.567–6.557	2.635	0.784–8.850
**Practice experience**				
2–10 years	Ref.		Ref.	
11–15 years	0.973	0.494–1.919	1.247	0.625–2.488
16–20 years	0.443	0.187–1.048	1.335	0.565–3.155
21–25 years	0.407	0.166–0.998 *	0.973	0.397–2.385
26+ years	0.416	0.141–1.221	0.845	0.283–2.522
**Number of patients with known risk factors**				
0–20	Ref.		Ref.	
21–50	0.619	0.354–1.084	0.945	0.548–1.631
51–100	0.752	0.412–1.370	2.050	1.115–3.769 *
More than 100	0.628	0.306–1.285	1.174	0.584–2.361
**Major region practising in**				
Lake Geneva region	Ref.		Ref.	
Espace Mittelland	0.267	0.089–0.796 *	0.601	0.205–1.760
North–West Switzerland	0.435	0.115–1.644	0.190	0.051–0.709 *
Zurich	0.463	0.130–1.647	0.273	0.079–0.944 *
Eastern Switzerland	0.133	0.035–0.510 **	0.183	0.049–0.680 *
Central Switzerland	0.345	0.092–1.287	0.329	0.090–1.209
**Main practising language**				
German	Ref.		Ref.	
French	0.389	0.126–1.207	0.137	0.045–0.421 **
**Vaccination attitudes**				
Confidence	1.009	0.933–1.091	1.074	0.989–1.167
Calculation	1.039	0.956–1.129	0.993	0.911–1.083
N	300		300	

* *p* < 0.05; ** *p* < 0.01; aOR: adjusted odds ratio; CI: confidence interval.

## Data Availability

The data presented in this study are openly available on Open Science Framework at https://osf.io/a84mj/ (accessed on 25 April 2023).

## Supplementary Materials

The following supporting information can be downloaded at: https://www.mdpi.com/article/10.3390/vaccines11061101/s1, Table S1. Reclassification of the 26 Swiss cantons into seven major statistical regions of Switzerland; Table S2. Ordinal logistic regression on perceived complexity of vaccination recommendations [1;4] (*n* = 300); Table S3. Ordinal logistic regressions on attitudes towards pneumococcal disease for adult patients with known risk; Figure S1. Patient flow through the study; Figure S2. Distribution of scores in the 5C measure of psychological antecedents of vaccination (*n* = 300); Figure S3. Risk groups GPs were not aware of (*n* = 172); Figure S4. Perception and attitudes about pneumococcal vaccination (*n* = 300); Figure S5. Knowledge and attitudes towards pneumococcal disease in adult risk patients (*n* = 300); Physician questionnaire.
