# Supplementary material for: General Practitioners’ Awareness and Perception of Current Pneumococcal Vaccination for Adult Patients with Known Risk Factors in Switzerland: Evidence from a Survey
Source: Vaccines (Basel). 2023 Jun 15;11(6):1101. doi: 10.3390/vaccines11061101 (PMC10302954; doi:10.3390/vaccines11061101)
Supplement: Supplementary file 1 [file vaccines-11-01101-s001.zip › vaccines-2394418-supplementary.pdf]

Supplementary File 1

Table S1. Reclassification of the 26 Swiss cantons into seven major statistical regions of Switzerland.

Major statistical regions	Cantons
Central Switzerland	Lucerne, Nidwalden, Obwalden, Schwyz, Uri, Zug
Eastern Switzerland	Appenzell Ausserrhoden, Appenzell Innerhoden, Glarus, Grisons, Schaffhausen, , St. Gallen, Thurgau
Espace Mittelland	Berne, Fribourg, Solothurn, Neuchâtel, Jura
Lake Geneva	Geneva, Valais, Vaud
North-West Switzerland	Aargau, Basel-Stadt, Basel-Landschaft
Ticino	Ticino
Zurich	Zurich

Table S2. Ordinal logistic regression on perceived complexity of vaccination recommendations [1;4] (N = 300).

	Unadjusted model		Adjusted model	
**Variable**	OR	95% CI	aOR	95% CI
**Gender**				
Male	Ref.		Ref.	
Female	1.228	0.799 - 1.888	1.299	0.817 - 2.064
**Age**				
40 or younger	Ref.		Ref.	
41-45	0.822	0.372 - 1.819	0.804	0.352 - 1.836
46-50	1.423	0.673 - 3.010	1.483	0.651 - 3.375
51-55	0.926	0.447 - 1.918	0.771	0.304 - 1.957
56-60	0.824	0.388 - 1.750	0.882	0.313 - 2.482
61 or older	1.368	0.620 - 3.017	1.391	0.436 - 4.432
**Practice experience**				
2-10 years	Ref.		Ref.	
11-15 years	0.893	0.485 - 1.644	0.833	0.421 - 1.649
16-20 years	1.309	0.666 - 2.574	1.483	0.632 - 3.480
21-25 years	0.702	0.381 - 1.292	0.677	0.284 - 1.614
26+ years	1.216	0.633 - 2.337	1.018	0.358 - 2.899
**Number of patients with known risk factors**				
0-20	Ref.		Ref.	
21-50	0.786	0.460 - 1.344	0.862	0.497 - 1.497
51-100	0.749	0.414 - 1.353	0.744	0.404 - 1.369
More than 100	0.774	0.386 - 1.553	0.761	0.373 - 1.552
**Major region practising in**				
Lake Geneva region	Ref.		Ref.	
Espace Mittelland	1.322	0.673 - 2.595	1.717	0.608 - 4.852
North-West Switzerland	0.846	0.405 - 1.767	1.098	0.301 - 4.009
Zurich	1.390	0.708 - 2.730	1.949	0.563 - 6.750
Eastern Switzerland	0.702	0.332 - 1.484	0.825	0.225 - 3.020
Central Switzerland	1.313	0.629 - 2.741	1.883	0.519 - 6.827
**Practising language**				
German	Ref.		Ref.	
French	1.036	0.633 - 1.696	1.293	0.436 - 3.836
**Vaccination attitudes**				
Confidence	0.973	0.905 - 1.045	0.969	0.897 - 1.047
Calculation	0.978	0.903 - 1.060	0.958	0.878 - 1.044
N	300		300	

* $p < 0.05$; ** $p < 0.01$, aOR: adjusted odds ratio; CI: confidence interval.

Table S3. Ordinal logistic regressions on attitudes towards pneumococcal disease for adult patients with known risk.

	I think that the pneumococcal disease is serious enough to justify vaccination [1;7]		Pneumococcal disease can cause considerable morbidity and mortality [1;7]		Pneumococcal vaccination is even more important during the COVID-19 pandemic [1;7]		Simplification of vaccination guidelines would result in more at-risk patients being vaccinated [1;7]		The pneumococcal disease has a mild disease course [1;7]		The risk of pneumococcal disease is low [1;7]		I am worried about the potential side effects of the pneumococcal vaccine [1;7]		Pneumococcal vaccination should mainly target children because of herd immunity [1;7]	
**Variable**	aOR	95% CI	aOR	95% CI	aOR	95% CI	aOR	95% CI	aOR	95% CI	aOR	95% CI	aOR	95% CI	aOR	95% CI
**Gender**																
Male	Ref.		Ref.		Ref.		Ref.		Ref.		Ref.		Ref.		Ref.	
Female	1.783	1.071 - 2.969*	1.046	0.653 - 1.677	1.205	0.757 - 1.919	1.298	0.824 - 2.043	0.793	0.495 - 1.269	0.713	0.452 - 1.123	0.752	0.474 - 1.193	1.298	0.811 - 2.076

**Age**																
40 or younger	Ref.		Ref.		Ref.		Ref.		Ref.		Ref.	1.000 - 1.000	Ref.		Ref.	
41-45	0.982	0.396 - 2.437	0.360	0.151 - 0.860*	0.911	0.397 - 2.089	1.495	0.682 - 3.279	1.447	0.613 - 3.418	1.204	0.521 - 2.784	1.169	0.504 - 2.713	1.427	0.612 - 3.325
46-50	1.343	0.526 - 3.427	0.609	0.254 - 1.462	0.725	0.313 - 1.677	1.680	0.744 - 3.794	1.161	0.484 - 2.785	0.948	0.410 - 2.194	0.864	0.362 - 2.058	0.992	0.420 - 2.341
51-55	1.108	0.404 - 3.043	0.410	0.159 - 1.057	0.966	0.384 - 2.430	0.979	0.399 - 2.401	1.397	0.536 - 3.637	0.942	0.373 - 2.381	0.935	0.360 - 2.427	0.874	0.335 - 2.280
56-60	1.387	0.452 - 4.253	0.370	0.133 - 1.030	0.912	0.333 - 2.498	0.808	0.301 - 2.169	1.119	0.391 - 3.202	1.030	0.375 - 2.831	0.730	0.255 - 2.089	1.153	0.405 - 3.286
61 or older	1.794	0.504 - 6.392	0.763	0.231 - 2.519	1.261	0.407 - 3.902	1.469	0.470 - 4.597	1.066	0.329 - 3.458	0.877	0.285 - 2.695	0.527	0.164 - 1.698	0.850	0.265 - 2.731
**Practice experience**																
2-10 years	Ref.		Ref.		Ref.		Ref.		Ref.		Ref.		Ref.		Ref.	
11-15 years	0.784	0.368 - 1.668	1.129	0.560 - 2.276	1.331	0.669 - 2.650	0.984	0.504 - 1.923	1.171	0.580 - 2.365	1.258	0.645 - 2.455	1.379	0.686 - 2.773	1.233	0.618 - 2.462
16-20 years	0.912	0.359 - 2.321	0.700	0.295 - 1.659	1.574	0.678 - 3.651	1.244	0.529 - 2.924	1.154	0.491 - 2.711	1.273	0.556 - 2.917	0.941	0.394 - 2.245	1.188	0.490 - 2.881
21-25 years	0.608	0.238 - 1.551	0.979	0.421 - 2.280	1.241	0.531 - 2.900	0.959	0.406 - 2.270	0.942	0.401 - 2.213	0.726	0.315 - 1.675	1.273	0.538 - 3.010	1.420	0.586 - 3.439
26+ years	0.345	0.111 - 1.076	0.385	0.137 - 1.083	0.738	0.272 - 2.004	0.417	0.150 - 1.163	1.787	0.630 - 5.068	1.666	0.622 - 4.461	4.644	1.648 - 13.083**	1.509	0.527 - 4.315
**Number of patients with known risk factors**																
0-20	Ref.		Ref.		Ref.		Ref.		Ref.		Ref.		Ref.		Ref.	
21-50	1.142	0.626 - 2.084	1.543	0.882 - 2.699	0.677	0.390 - 1.177	0.942	0.552 - 1.606	0.996	0.561 - 1.769	0.945	0.544 - 1.641	0.581	0.329 - 1.025	0.601	0.340 - 1.063
51-100	1.579	0.801 - 3.112	1.696	0.909 - 3.166	1.282	0.692 - 2.375	0.810	0.447 - 1.468	0.690	0.368 - 1.292	0.757	0.414 - 1.387	0.658	0.359 - 1.205	0.814	0.441 - 1.504
More than 100	1.796	0.825 - 3.912	2.620	1.252 - 5.485*	2.076	1.008 - 4.273*	0.657	0.325 - 1.331	0.877	0.426 - 1.808	0.623	0.306 - 1.271	0.648	0.321 - 1.305	0.259	0.123 - 0.546**
**Major region practising in**																
Lake Geneva region	Ref.		Ref.		Ref.		Ref.		Ref.		Ref.		Ref.		Ref.	
Espace Mittelland	0.263	0.080 - 0.864*	0.514	0.175 - 1.507	0.355	0.120 - 1.049	1.573	0.517 - 4.783	2.649	0.813 - 8.633	3.672	1.240 - 10.876*	0.784	0.269 - 2.285	3.546	1.123 - 11.196*
North-West Switzerland	0.409	0.094 - 1.777	0.490	0.126 - 1.899	0.308	0.079 - 1.202	1.107	0.286 - 4.292	1.826	0.442 - 7.541	3.271	0.862 - 12.407	0.522	0.138 - 1.973	1.983	0.490 - 8.017
Zurich	0.458	0.114 - 1.835	0.481	0.135 - 1.717	0.548	0.155 - 1.947	1.261	0.351 - 4.528	2.792	0.720 - 10.828	3.954	1.101 - 14.207*	0.666	0.188 - 2.360	2.374	0.629 - 8.965
Eastern Switzerland	0.346	0.081 - 1.474	0.338	0.089 - 1.285	0.558	0.146 - 2.126	1.096	0.291 - 4.123	4.573	1.105 - 18.916*	5.077	1.342 - 19.204*	1.178	0.310 - 4.472	3.528	0.886 - 14.051
Central Switzerland	0.298	0.071 - 1.250	0.289	0.076 - 1.093	0.467	0.126 - 1.736	0.876	0.235 - 3.273	4.426	1.086 - 18.042*	4.883	1.304 - 18.283*	0.546	0.147 - 2.030	2.852	0.726 - 11.208
**Practising language**																
German	Ref.		Ref.		Ref.		Ref.		Ref.		Ref.		Ref.		Ref.	
French	0.941	0.281 - 3.149	1.031	0.338 - 3.150	0.713	0.232 - 2.195	2.755	0.873 - 8.697	0.839	0.256 - 2.744	1.555	0.513 - 4.713	0.500	0.165 - 1.516	0.917	0.287 - 2.930
**Vaccination attitudes**																
Confidence	1.391	1.263 - 1.531**	1.214	1.118 - 1.318**	1.180	1.088 - 1.280**	1.079	0.997 - 1.167	0.886	0.821 - 0.956**	0.925	0.859 - 0.995*	0.847	0.782 - 0.917**	0.936	0.867 - 1.010
Calculation	1.160	1.053 - 1.277**	1.024	0.938 - 1.118	1.134	1.034 - 1.243**	1.075	0.988 - 1.170	0.994	0.911 - 1.084	0.939	0.862 - 1.023	1.017	0.930 - 1.112	0.904	0.827 - 0.988*
N	300		300		300		300		300		300		300		300	

* p <0.05; ** p < 0.01; aOR: adjusted odds ratio; CI: confidence interval.

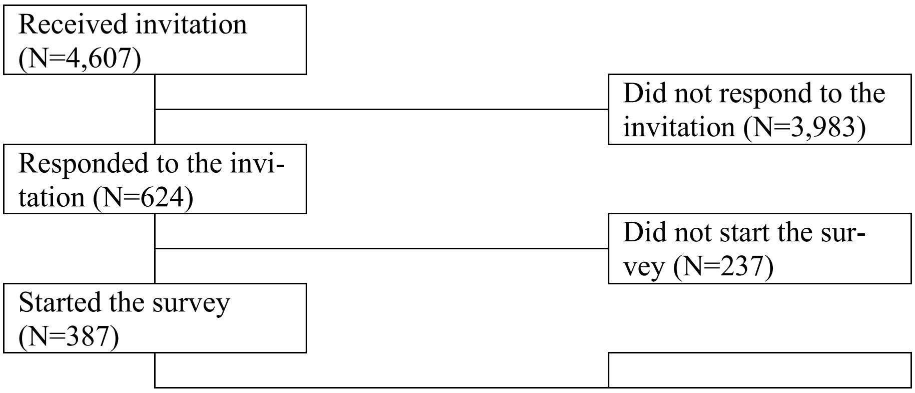

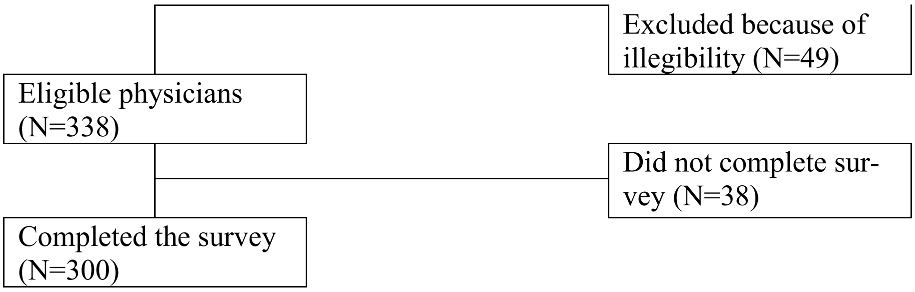

Figure S1. Patient flow through the study.

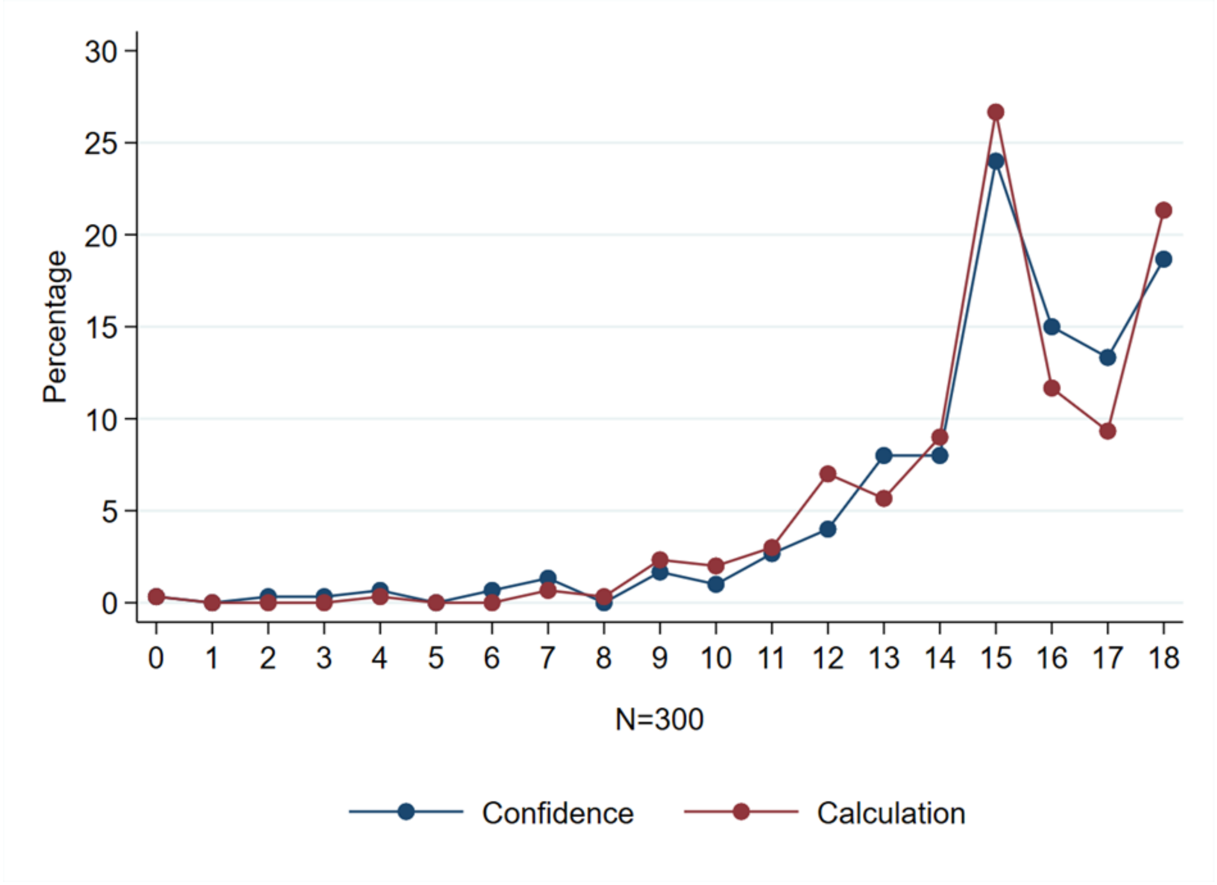

Figure S2. Distribution of scores in the 5C measure of psychological antecedents of vaccinations (N = 300).

Note: Confidence is defined as trust in the effectiveness and safety of vaccines and the health system delivering them, including the reliability and competence of the health services and health professionals and the motivations of vaccination policies. Individuals with high confidence have positive attitudes towards vaccination. Calculation refers to individuals' engagement in extensive information searching. A high calculation score indicates that the individuals evaluate risks of infections and vaccination to achieve a good decision.

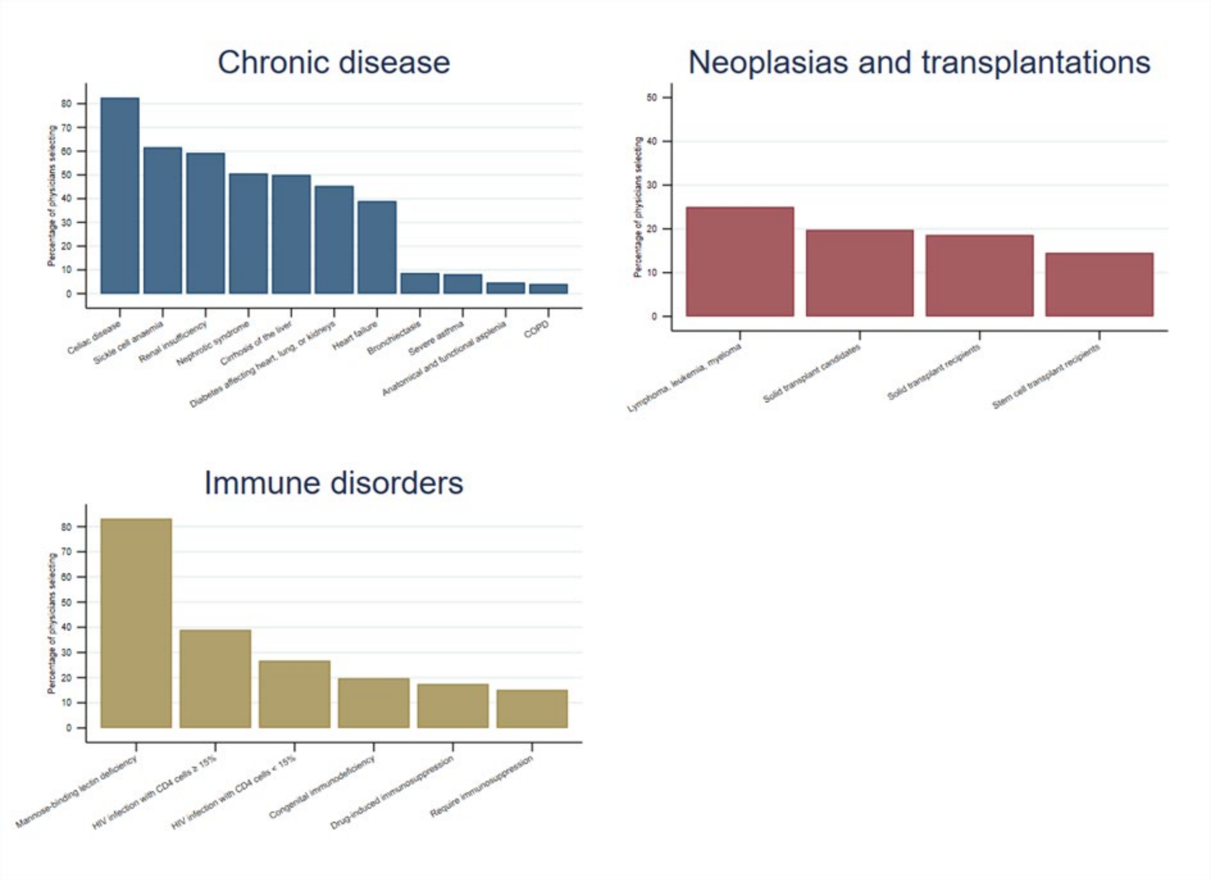

Figure S3. Risk groups GPs were not aware of (N=172).

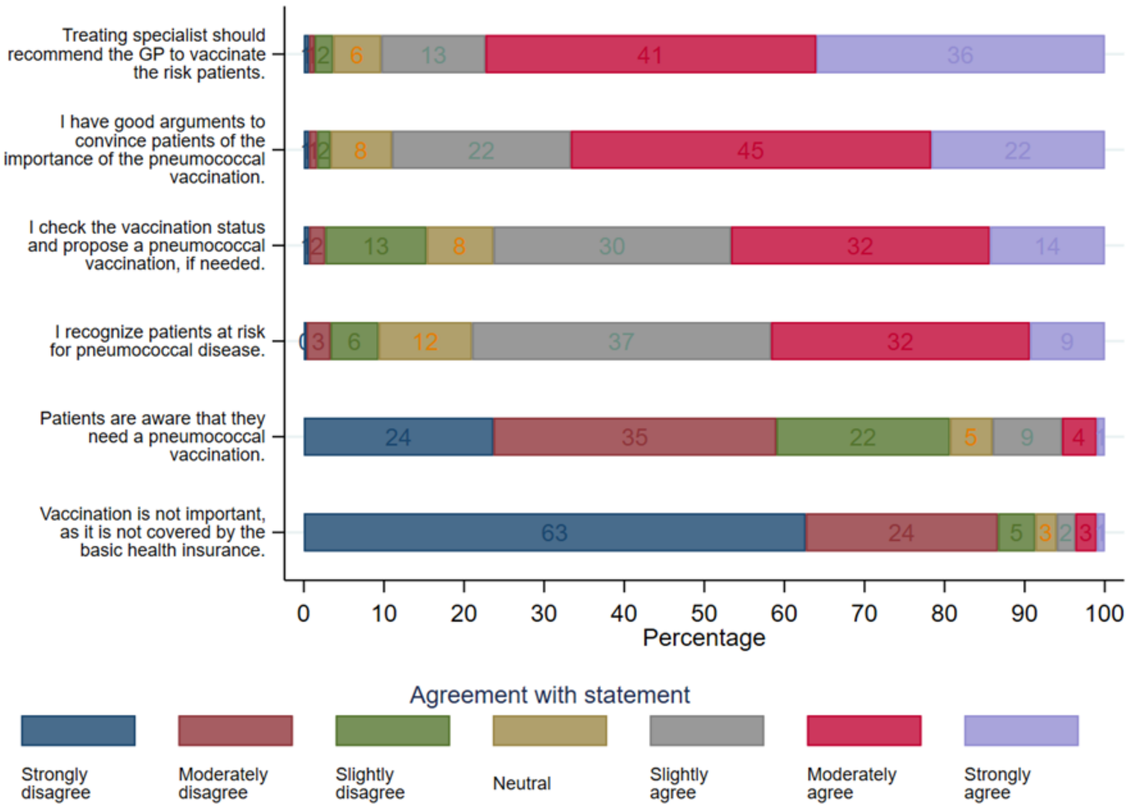

Figure S4. Perceptions and attitudes about pneumococcal vaccination (N = 300).

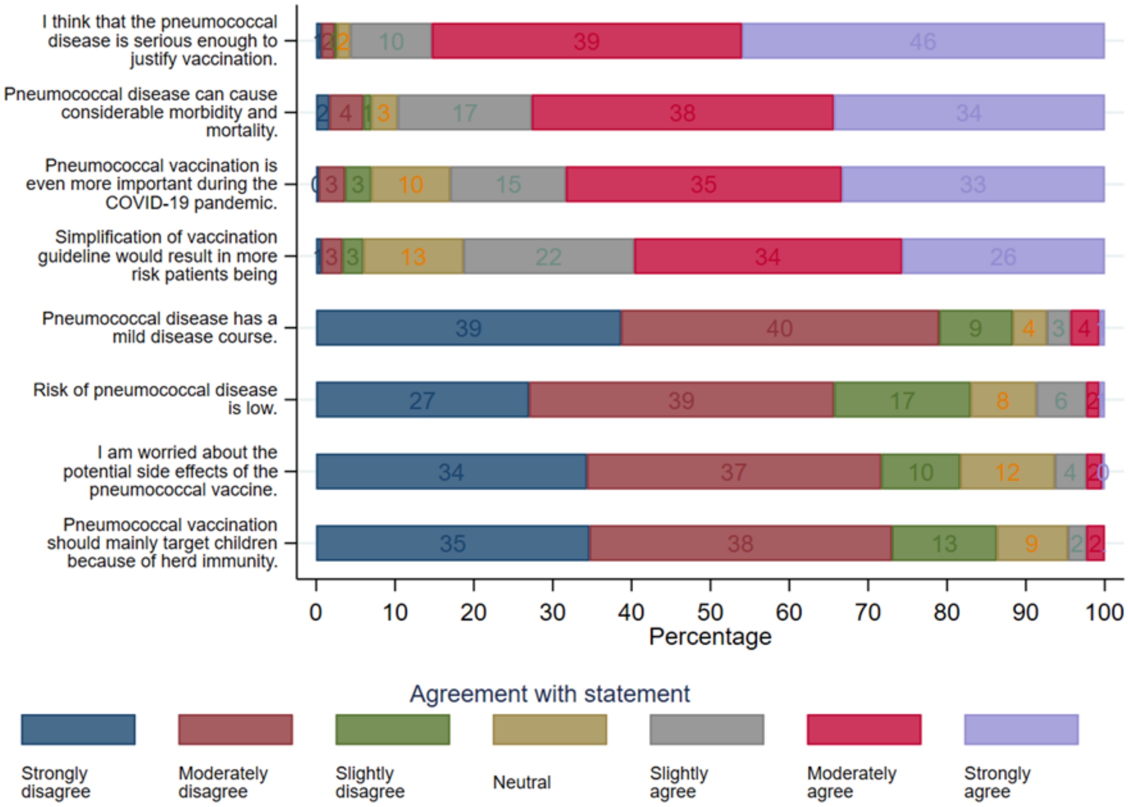

Figure S5. Knowledge and attitudes towards pneumococcal disease in adult risk patients (N = 300).
